# Blue-Print Autophagy: Potential for Cancer Treatment

**DOI:** 10.3390/md14070138

**Published:** 2016-07-21

**Authors:** Nadia Ruocco, Susan Costantini, Maria Costantini

**Affiliations:** 1 Department of Biology and Evolution of Marine Organisms, Stazione Zoologica Anton Dohrn, Villa Comunale, 80121 Napoli, Italy; nadia.ruocco@szn.it; 2 Department of Biology, University of Naples Federico II, Complesso Universitario di Monte Sant’Angelo, Via Cinthia, 80126 Napoli, Italy; 3 Bio-Organic Chemistry Unit, Institute of Biomolecular Chemistry-CNR, Via Campi Flegrei 34, Pozzuoli, 80078 Naples, Italy; 4 CROM, Istituto Nazionale Tumori “Fondazione G. Pascale”, IRCCS, 80131 Napoli, Italy; s.costantini@istitutotumori.na.it

**Keywords:** autophagy, cancer, marine environment, natural products

## Abstract

The marine environment represents a very rich source of biologically active compounds with pharmacological applications. This is due to its chemical richness, which is claiming considerable attention from the health science communities. In this review we give a general overview on the marine natural products involved in stimulation and inhibition of autophagy (a type of programmed cell death) linked to pharmacological and pathological conditions. Autophagy represents a complex multistep cellular process, wherein a double membrane vesicle (the autophagosome) captures organelles and proteins and delivers them to the lysosome. This natural and destructive mechanism allows the cells to degrade and recycle its cellular components, such as amino acids, monosaccharides, and lipids. Autophagy is an important mechanism used by cells to clear pathogenic organism and deal with stresses. Therefore, it has also been implicated in several diseases, predominantly in cancer. In fact, pharmacological stimulation or inhibition of autophagy have been proposed as approaches to develop new therapeutic treatments of cancers. In conclusion, this blue-print autophagy (so defined because it is induced and/or inhibited by marine natural products) represents a new strategy for the future of biomedicine and of biotechnology in cancer treatment.

## 1. Introduction to Autophagy

Over the last fifty years, research on programmed cell death has evolved very rapidly because it represents a key point in cellular housekeeping processes. In fact, aberrant cell death regulation leads to several diseases, including various autoimmune diseases, cancer, stroke, myocardial infarctations, and neurodegenerative diseases [1,2,3,4]. The programmed cell death process can be subdivided into three different categories: oncosis (also named necrosis), apoptosis, and autophagy or lysosomal cell death [5,6,7]. These three different types are based on the cellular signaling pathways involved in the process and on the morphology of the dying cells. Oncosis, or “accidental cell death”, is characterized by cellular swelling followed by cell membrane bursting and the release of inflammatory signals [8]. It is most commonly triggered from acute cellular damage, from severe cytotoxicity, or from a failure of the ionic pumps within the plasma membrane. Apoptosis represents an evolutionary well-conserved form of cell suicide, coordinated by members of the caspase family of cysteine proteases. It is defined by characteristic morphological features, which include cell shrinkage, chromatin condensation, membrane blebbing, and internucleosomal DNA fragmentation [9,10]. Autophagy further distinguishes itself from apoptosis by inducing the degradation of organelles at an early stage of cell death, but preserving the intactness of the cytoskeleton until the final phase of the dying process [6,11]. In many aspects autophagy is very closely related to apoptosis, but differs from apoptosis by being a mainly caspase-independent process. However, even if apoptosis is the most extensively studied form of programmed cell death, autophagy has recently aroused an enormous research interest [12,13].

**Figure 1:**
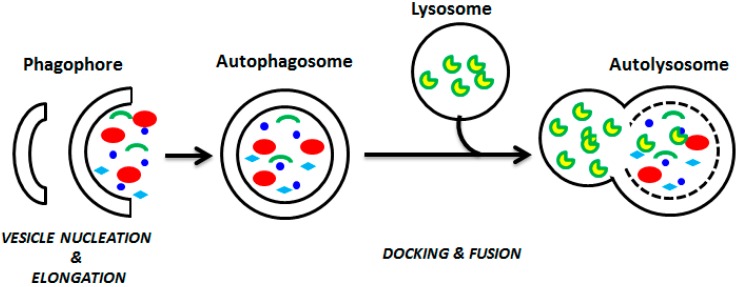
Schematic diagram of the steps of autophagy. Autophagy begins with the formation of the phagophore (vesicle nucleation step), which leads to the expansion of the phagophore into an autophagosome (vesicle elongation) with the helping of specific proteins. The autophagosome contains some different damaged organelles (shown by different colors), which can fuse with a lysosome (docking and fusion steps) forming an autolysosome.

For this reason, in this review we focused our attention on the autophagy process (Figure 1).

In detail, autophagy is the sequential process by which the cytoplasmic components are engulfed by a phagophore, isolated in autophagosome, and broken down in the lysosome [14,15]. It is triggered primarily by fasting [6], by endoplasmic reticulum stress resulting from an accumulation of misfolded proteins [16], and by various other genotoxic stress factors [11]. The eukaryotic initiation factor 2α (eIF2α) and the 5′-AMP-activated protein kinase (AMPK) are considered the major cellular inducers of autophagy, activated in response to starvation, to endoplasmic reticulum stress, or to low cellular energy levels [13]. Other cellular inducers of autophagy are the tumor suppressor protein p53, the endoplasmic reticulum membrane-associated protein Ire-1, and calcium [12]. Proteins belonging to the autophagy-related family (Atg) are involved in the downstream execution of autophagy, by which it is possible to mediate the maturation and the expansion of autophagosomes. Lysosomal transmembrane proteins assist with the fusion of the autophagosomes with lysosomes, and various lysosomal cysteine proteases ensure the degradation of autophagosomal contents. The TOR (target of rapamycin) kinase is the major cellular inhibitor of autophagy, repressing autophagy in response to growth factor and insulin-like signals during nutrient abundance [17,18].

**Figure 2:**
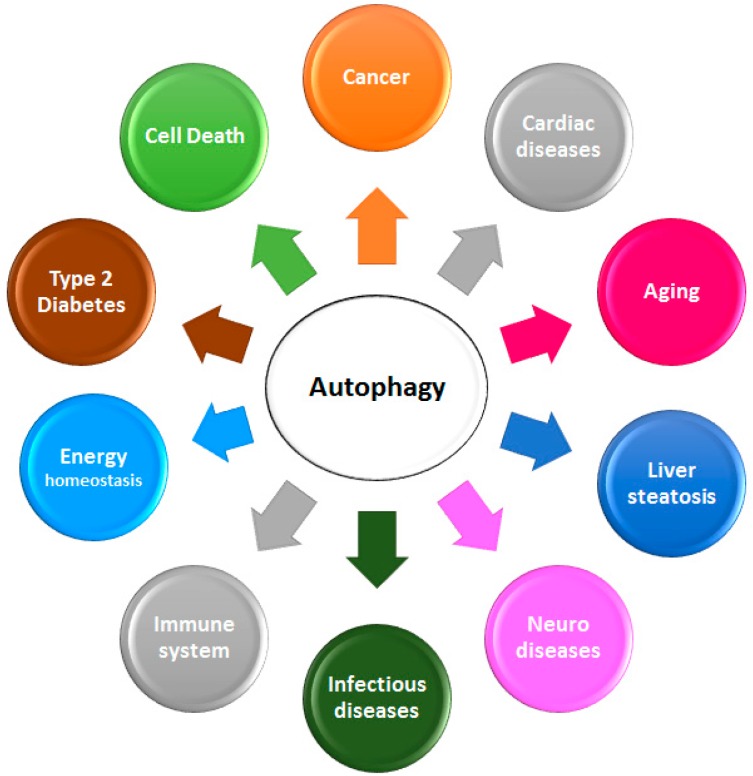
Involvement of autophagy in different pathological and physiological processes.

Autophagy is also considered to be a well-regulated cell survival mechanism. In fact, it plays a crucial role in the maintenance of normal human physiological processes, including cellular homeostasis, energetic balance, and development [19,20,21]. Autophagy has numerous pathological and physiological functions, and is implicated in pathological and physiological processes such as energy homeostasis, neurodegenerative diseases, type 2 diabetes, infectious diseases, cardiomyopathy, and diseases linked to aging and innate immune system, including cancer (Figure 2; [6,22,23,24,25]).

In this review we give a general overview on autophagy, and how this process—induced by marine natural products—represents a new strategy for the future of biomedicine and of biotechnology in cancer treatment.

## 2. Autophagy and Cancer

Since autophagy is able to act in response to inflammatory reactions [14], metabolic requirements [26], and on the oxidative stress [27], it plays different roles in cancer [22,28,29,30,31], enhancing cancer development [15,32]. In more detail, autophagy has dual roles in cancer, acting as both (i) a tumor suppressor, by preventing the accumulation of damaged proteins and organelles; and (ii) as a mechanism of cell survival, promoting the growth of established tumors. Tumor cells activate autophagy in response to cellular stress and/or increased metabolic demands related to rapid cell proliferation. Autophagy-related stress tolerance can enable cell survival by maintaining energy production, which can lead to tumor growth and therapeutic resistance. Autophagy defects are associated with susceptibility to metabolic stress, genomic damage, and tumorigenesis in mice, indicating a role for autophagy in tumor suppression [33].

Autophagy is a homeostatic, catabolic degradation process whereby cellular proteins and organelles are engulfed by autophagosomes, digested in lysosomes, and recycled to sustain cellular metabolism.

A major regulator of autophagy is the mammalian target of rapamycin (mTOR) pathway, which consists of two distinct signaling complexes known as mTORC1 and mTORC2 [34]. mTOR is activated downstream of PI3KAKT, a pathway that is commonly dysregulated in human cancer [35,36]. Cellular stress leads to downregulation of mTOR1 activity that triggers autophagy [34], and in this regard, mTOR inhibitors, including rapamycin, have been shown to induce autophagy in tumor cells [35,37].

Autophagy can be induced by hypoxia, a stimulus for AMPK, which is mediated by hypoxia-inducible factor (HIF) and its target gene BNIP3 [36]. The tumor suppressor p53 protein can modulate autophagy by its cellular localization [38].

Evidence indicates that the predominant role of autophagy in cancer cells is to confer stress tolerance, which serves to maintain tumor cell survival [14,31]. Knockdown of essential autophagy genes in tumor cells has been shown to confer or potentiate the induction of cell death [39,40]. Cytotoxic and metabolic stresses, including hypoxia and nutrient deprivation, can activate autophagy for recycling of ATP for maintaining cellular biosynthesis and survival. Autophagy is induced in hypoxia by HIF-1α-dependent and -independent mechanisms [41].

As reported above, autophagy also acts as a tumor suppression mechanism by removing damaged organelles/proteins and limiting cell growth and genomic instability [33,41]. Excessive stimulation of autophagy due to Beclin1 overexpression can inhibit tumor development [42]. A potential molecular link between defective autophagy and tumorigenesis involves the accumulation of p62/SQSTM 1 protein aggregates, damaged mitochondria, and misfolded proteins that lead to the production of reactive oxygen species (ROS). This causes DNA damage, leading to genomic instability [40]. Autophagy may also protect against tumorigenesis by limiting necrosis and chronic inflammation, which are associated with the release of pro-inflammatory HMGB1 [43]. Together, these findings establish a role for autophagy as a mechanism of tumor suppression.

In addition to the cytoprotective function of autophagy, induction of autophagic cell death has been proposed as a mechanism of cell death, given that features of autophagy have been observed in dying cells. In cancer cells, autophagy accompanied by non-apoptotic cell death has been described [44,45]. Prolonged stress and sustained autophagy may eventually lead to cell death when protein and organelle turnover overwhelm the capacity of the cell. Induction of autophagic cell death by anticancer drugs may occur depending on the cell type and genetic background [46,47].

Hypoxic microenvironment is very often the place where tumor cells reside, suggesting that hypoxia and the resulting tumor acidity can be involved in cancer progression, mainly in tumor resistance to chemotherapy [48,49]. For these reasons, in these unfavorable acidic conditions, compensatory mechanisms have been developed by tumor cells, which are able to confer survival and growth advantages [48]. A very important key role in the unfavorable tumor microenvironment is attributed to the vacuolar ATPases (v-ATPases) [48,49]. This enzyme family utilizes energy freed by the hydrolysis of ATP to pump protons from the cytoplasm to the lumen of intracellular compartments in the cytoplasm, for example to the lysosomes in order to maintain an alkaline pH [50,51]. Through this mechanism, tumor cells free themselves from H^+^ ions, which otherwise accumulate in the cytoplasm and stimulate lytic enzymes active at low pH [49]. In some malignant cells, such as pancreatic cancer cells, v-ATPases are also located in the plasma membrane to pump protons from the cytoplasm to the exterior [49,52], creating an acidic tumor microenvironment important for cancer initiation and progression. Because of this, v-ATPases are considered potential therapeutic targets in cancer [49]. Moreover, v-ATPases also play a role in chemoresistance of cancer cells, thanks to the neutralization of weakly basic drugs by the acidic tumor microenvironment [49,51]. In established hypoxic tumors, for example pancreatic ductal adenocarcinoma (PDAC), autophagy is up-regulated and tumor cells use its catabolic function in order to tolerate stress [53]. In pancreatic cancer cells, transformation by oncogenic K-Ras may induce basal autophagy, which is required in these cells to maintain energy balance for tumor growth. Thus, drugs able to inactivate autophagic proteolysis may have a unique potential to treat cancers such PDAC [53], or to sensitize them for chemotherapeutic treatments.

Evidences show a crosstalk between autophagy and apoptosis. When autophagy is inhibited, apoptosis is promoted in cancer cells with intact apoptotic signaling [38]. Disabled apoptosis is a frequent occurrence in human cancers, and tumors under stress generally die by other cell-death mechanisms. Autophagy may be an alternative mode of cell death in apoptosis-resistant cells [54,55]. This cross-talk between autophagy and apoptosis exists at many levels because both pathways share mediators, ranging from the core machinery to upstream regulators [54]. Recent findings suggest a link between autophagy and the extrinsic apoptotic pathway that is mediated by p62/SQSTM 1 [56] and by Bcl-2 family proteins [57].

## 3. Autophagy and Marine Natural Products

Several studies have demonstrated the importance of natural products as sources of new drugs in the last 25 years. Particularly, 47% of anticancer drugs are of natural origin or directly derived from nature, and up to 70% could be considered structurally related to natural compounds [58]. The demand and need of new drugs is becoming continuous and important for public health, because of the increase in the incidence of severe diseases such as cancer [59,60]. This urgent need to discover new drug entities is pushing the researcher to explore the marine environment. In fact, many new interesting compounds, which are commonly referred to as marine natural products, have been discovered in the last few years [61]. The chemical diversity of natural compounds from marine organisms could be due to the specific environmental conditions of the sea, such as high pressure and salt content as well as varying pH values. Moreover, the marine environment includes a number of macro and micro-organisms (such as bacteria, cyanobacteria, fungi, algae, microalgae, or small invertebrates), which have developed chemical compounds as special defense strategies to support their survival in diverse, competitive, and hostile habitats [62,63]. In fact, marine organisms are able to produce a large variety of small molecular substances with specific bioactive characteristics. Not surprisingly, these substances have moved into the focus of cancer research in the past decades. Indeed, marine drugs, such as cytarabin (Ara-C), trabectedin, and eribulin, are used clinically to treat different malignancies including leukemias, lymphomas, soft tissue sarcomas, and breast cancer [64,65,66,67,68].

In the last decades the research field of marine natural products, aiming to identify potent anti-cancer compounds among the toxins produced by marine organisms as a chemical defense mechanism against their predators, represents a promising field [69]. The major bio-products obtained from marine organisms include carotenoids, fatty acids, glycolipids, polysaccharides, and proteins, with potential interest in the treatment and prevention of inflammatory diseases and cancer. Many marine organisms, such as sessile invertebrates lacking physical defense mechanisms, have developed potent toxins as chemical defenses against their predators. The toxins produced by marine organisms display unique chemical and biological features and have promising cytotoxic effects on cancer cells [70,71]. To date, several of the numerous cytotoxic marine natural products identified have been described as activators of the different types of programmed cell death [72,73,74]. This was not considered a surprising result, taking into account that caspase-mediated apoptosis represents an evolutionary highly conserved process. In fact, from an ecological or evolutionary perspective this process has already been found in basal metazoan phyla such as sponges and cnidarians and even in pre-metazoan phytoplankton [13]. Furthermore, apoptosis plays critical roles in the metamorphosis of sea urchins [75] and of ascidians [76], and is involved in various host/parasite interactions [77]. Autophagy protects mussels [78] and sea cucumbers [79] against various environmental stresses. Autophagy has severe ecological implications in coral bleaching [80]. Several studies reported the role of marine natural products as autophagy inhibitors and inducers isolated from several species, such as marine sponges, sea urchins, algae, cyanobacteria, bacteria, and fungi [81,82,83,84]. In the following paragraphs we describe the effects of natural products isolated from marine organisms on the autophagy pathways, also discussing their applications in biomedicine and biotechnology.

### 3.1. Autophagy-Inducers Marine Natural Products

**Figure 3:**
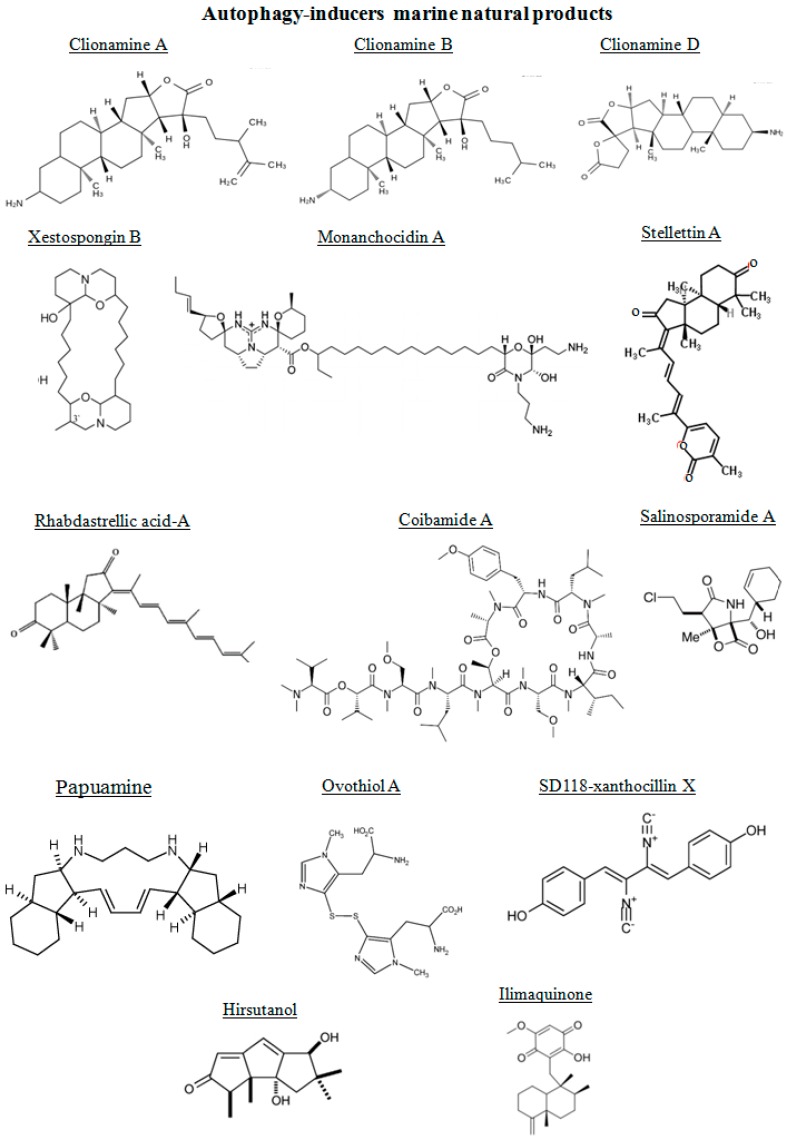

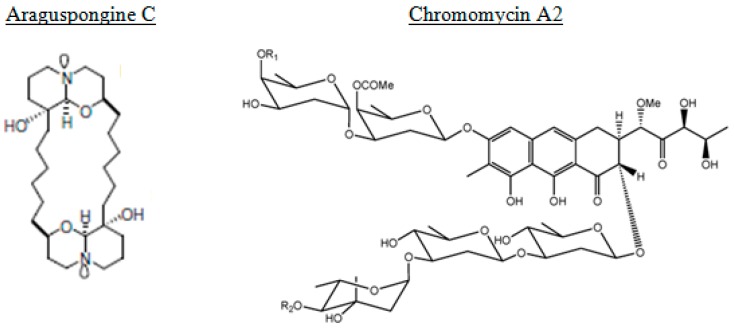
Chemical structure of different autophagy-inducers, natural products from marine organisms.

The chemical structures of the marine autophagy-inducers marine natural products are reported in the Figure 3. Four 3β-aminosteroids isolated from the marine sponge *Cliona celata* are involved in stimulating autophagy: clionamines A, B, C and D [85]. The clionamines contain structural features not previously encountered in naturally occurring steroids. They are characterized by a combination of an E-ring γ-lactone and C-20 hydroxylation as in all of the analogues and the spirobislactone side chain found in clionamine D. Lam et al. [86], prompted by the need for novel small molecule modulators of autophagy as chemical tools and drug leads, screened a library of marine organism crude extracts in a cell-based high content assay designed to find both stimulators and inhibitors of autophagy. They found a MeOH extract of the sponge *C. celata* (collected on the Wild Coast of South Africa) with promising autophagy stimulation. The amino steroids clionamines A to D have been revealed by assay-guided fractionation of the extract, revealing that they were responsible for the biological activity [87]. The major component in the extract was clionamine A. Clionamines A to D induced autophagosome accumulation measured by the formation of cytoplasmic punctate green fluorescent protein (GFP)-LC3, an autophagy marker. This effect was increased in medium lacking amino acids and serum. Moreover, clionamine A caused a decrease in the level of GFP-LC3 and an increase in GFP revealed by immunoblotting. These results indicated that the 1A/1B-light chain 3 (LC3) moiety of the fusion protein was degraded and that clionamine A stimulates autophagy, particularly under starvation conditions. In order to generate sufficient quantities of a natural clionamine or a more potent analogue for in vivo studies in animal models, Forestieri et al. [85] synthetized the clionamine B starting from the plant saponigen tigogenin. This synthetic clionamine B strongly stimulated autophagy in human estrogen-responsive breast cancer MCF7 cells.

The alkaloid xestospongin B, a macrocyclic bis-1-oxaquinolizidine alkaloid isolated from the sponge *Xestospongia exigua*, has been shown to induce autophagy through its inositol triphosphate receptor antagonistic properties in neuroblastoma (NG108-15) cells [88,89].

From the marine sponge *Xestospongia* species, araguspongines have also been isolated [90]. They represent a group of macrocyclic oxaquinolizidine alkaloids. The anticancer activity of the known oxaquinolizidine alkaloids araguspongines A, C, K, and L were evaluated against breast cancer cells. Araguspongine C inhibited the proliferation of multiple breast cancer cell lines in vitro in a dose-dependent manner. Furthermore, araguspongine C-induced autophagic cell death was observed in HER2-overexpressing BT-474 breast cancer cells, characterized by vacuole formation and upregulation of autophagy markers including LC3A/B, Atg3, Atg7, and Atg16L. Araguspongine C-induced autophagy was associated with suppression of c-Met and HER2 receptor tyrosine kinase activation [90].

Monanchocidin A (MonA) is a novel guanidine alkaloid with an unprecedented skeleton system derived from a polyketide precursor, (ω-3)-hydroxy fatty acid, and containing a 2-aminoethyl- and 3-aminopropyl-substituted morpholine hemiketal ring, isolated from the sponge *Monanhora pulchra*. MonA is a highly effective marine compound, offering a promising alternative in therapeutic activity of genitourinary cancers, where standard therapies fail. This property is due to the different mechanism of action in comparison with the standard chemotherapies. MonA effectively inhibited the growth of human leukemia cell lines HL-60 and THP-1, presumably by induction of apoptosis [91]. More recently, Dyshlovoy et al. [63] demonstrated that MonA exerted high cytotoxicity in human germ cell tumors (GCT), whereas non-malignant human cells were less sensitive. Moreover, the same activity of MonA has been revealed in castration-resistant and hormone-sensitive prostate cancer cell lines, as well as in cisplatin-resistant and -sensitive GCT cell lines, suggesting that MonA is active even if standard treatment regimens fail. MonA induced programmed cell death and arrest in G1- and S-phase cell cycle of GCT cells. Further experiments showed that the major mechanisms leading to cell death were the autophagy and/or lysosomal membrane permeabilization. Short-term treatment with high concentrations or long-term treatment with low concentrations of MonA induced unspecific degradation of various proteins. Furthermore, protein degradation induced by long-term treatment has been inhibited by the selective autophagy inhibitor 3-methyladenine [92]. In addition, autophagic vacuole formation after exposure to low concentrations of MonA and the up-regulation of the autophagy marker LC3B-II strongly suggest that the compound is an inductor of autophagy. In contrast, other cytotoxic substances, such as docetaxel or cisplatin, did not induce an up-regulation of LC3B-II in the human teratocarcinoma cell line NCCIT-R. This because their mechanism of action is the apoptosis and not autophagy.

Papuamine, a C2-symmetrical pentacyclic alkaloid from the marine sponge *Haliclona* sp., decreases survival of breast cancer MCF-7 cells, which when treated for four hours with papuamine revealed an increase in LC3 expression, suggesting that it was able to induce early autophagy in MCF-7 cells that later activated c-Jun *N*-terminal kinase (JNK) [93]. Stellettin A, a triterpene isolated from a marine sponge *Stelletta tenuis* [94], induced autophagy in B16F10 murine melanoma cells. An increase in LC3-II expression and its co-localization with tyrosinase indicated removal of deglycosylated and unfolded proteins [95].

Rhabdastrellic acid-A, an isomalabaricane triterpenoid purified from a marine sponge *Rhabdastrella globostellata*, induced autophagy in human lung cancer A549 cells. In Atg5 knockdown cells, rhabdastrellic acid-A mediated autophagy was impaired. pAkt was reduced in rhabdastrellic acid-A treated A549 cells and, interestingly, transfecting constitutively active Akt in A549 cells can inhibit rhabdastrellic acid-A induced autophagy [96].

Ilimaquinone, a sesquiterpene quinone, was originally isolated from the Hawaiian sponge, *Hippospongia metachromia* [97]. This compound promoted fragmentation of the Golgi apparatus through a microtubule-independent mechanism, thereby inhibiting vesicular protein transport, also activating hypoxia-inducible factor-1 (HIF-1). Moreover, it induced G2/M cell cycle arrest, apoptosis and autophagy, thereby exhibiting anti-proliferative activity in colon cancer cells with the wild-type p53 gene [97].

Algae represent another source of autophagy-inducers marine natural products. In fact, algal methanolic extracts derived from green alga *Enteromorpha intestinalis* and *Rhizoclonium riparium*, induced autophagy in HeLa cells, enhancing the expression of LC3-II [98]. From red seaweed *Laurencia dendroidea* the sesquiterpene elatol has been identified, with antiproliferative activity against *Leishmania amazonensis* with endoplasmic reticulum extension [99]. A carotenoid, the fucoxanthin, has been identified in edible brown algae. It showed dose-dependent cytotoxic activity and G0/G1 arrest of HeLa cells. Autophagy-based cytotoxicity of fucoxanthin-treated HeLa cells has been also found, acting as inhibitor of Akt/mTOR signaling pathway [100]. Coibamide A is an N-methyl-stabilized cyclopeptide natural product, named for its isolation from a marine cyanobacterium *Leptolyngbya* sp. collected from the Coiba National Park, Panama [101]. Coibamide A showed sub-nanomolar potency as a growth inhibitory agent in many of the tested cell lines, inducing concentration- and time-dependent cytotoxicity in human U87-MG and SF-295 glioblastoma cells and mouse embryonic fibroblasts (MEFs). The treatment with this compound induced a progressive loss of plasma membrane integrity and detachment of cells from culture dishes. This activity was lost upon linearization of the molecule, indicating the importance of the intact lariat macrocycle for inducing growth inhibitory and cytotoxic responses. These results have been greatly expanded by Hau et al. [102] clarifying the mechanism of action of the marine depsipeptide. Firstly, their results showed that coibamide A induces cell death with different signaling pathways according to cell type. SF-295 glioblastoma cells underwent caspase-3 activation and apoptosis in response to coibamide A, the same observed in wild-type and autophagy-deficient MEFs. Differently, U87-MG glioblastoma cells, more sensitive to coibamide A, underwent an alternate form of cell death through caspase inhibitor Z-VAD-FMK, with extensive cytoplasmic vacuolization and a lack of apoptotic features. Coibamide A induced two morphologically distinct forms of cell death in a cancer cell type that is typically resistant to treatment, facilitating further evaluation of this marine natural product as a potential lead compound.

A histidine-derived thiol has been isolated from sea urchin *Paracentrotus lividus* eggs, the ovothiol A disulfide. This compound has a key role in the protection of cells toward the oxidative burst associated with fertilization. The treatment of a human liver carcinoma cell line, Hep-G2, with ovothiol A induced a decrease of cell proliferation in a dose-dependent manner with a concomitant occurrence of autophagy, as revealed by phase contrast and fluorescence microscopy, together with the expression of the specific autophagic molecular markers, LC3 II and Beclin-1 [103]. These results shed light on ovothiol as a promising marine bioactive molecule able to inhibit cell proliferation in cancer cells.

Several other marine organisms have been considered a good source of bioactive natural compounds, such as coral, bacteria, and fungi. For example, hirsutanol, a sesquiterpene, has been isolated from marine fungus *Chondrostereum* sp. in the coral *Sarcophyton tortuosum* [104]. In breast cancer MCF-7 cells the treatment with hirsutanol increased the microtubule-associated protein 1A/1B-light chain 3 (LC3-I) to LC3-II conversion and ROS induction [105].

From marine bacteria *Salinispora tropica* and *Salinispora arenicola* has been isolated a potent proteasome inhibitor, the salinosporamide A. This marine natural compound induced autophagy through a phospho-eukaryotic translation initiation factor 2α (eIF2α) pathway so as to reduce proteotoxic stresses in human prostate cancer cells [106]. SD118-xanthocillin X, isolated from the marine fungus *Penicillium commune*, is an autophagy-inducer in hepatocellular carcinoma HepG2 cells. It regulated different modulators of autophagy by (i) exerting its autophagy-inducing effects via inhibition of phosphorylation of mTOR and ERK1/2 and/or (ii) attenuating the suppression of autophagy through inhibition of Bcl-2, an inhibitor of Beclin-1 [107].

Chromomycin A2 is a glycosylated tricyclic polyketide member of the aureolic acid group of antitumor antibiotics. Is has been isolated from a strain of *Streptomyces* sp. recovered from sediment collected at Paracuru Beach, located on the west coast of Ceará State, on the northeastern region of Brazil [108]. Guimaraes et al. [108] reported on evidences that show the induction of autophagy in melanoma cells by chromomycin A2, a feature not yet described for this class of compounds.

Information has also been found concerning other marine-derived agents, such as eicosapentaenoic acid (EPA) and docosahexaenoic acid (DHA), also potent inducers of autophagy as indicated by formation of autophagosomes in DHA- or EPA-treated lung adenocarcinoma A549 cells [109]. Finally, the Na+/K+-ATPases (NKA) inhibitor cardiac glycosides are a family of natural or synthetic steroid hormones isolated from marine or terrestrial natural products [110]. They exerted a potent anti-cancer property via activation of Src in the upstream of MEK1/2 and ERK1/2 pathway in human non-small cell lung cancer A549 and H460 cells [111]. Src inhibition, via the inhibitor PP2 or siRNA, remarkably repressed cardiac glycosides-induced MEK1/2 and ERK1/2 phosphorylation and autophagic cell death. Moreover, ROS were also accumulated, thereby contributing to cardiac glycosides-induced Src-mediated autophagic response in lung cancer cells.

### 3.2. Autophagy-Inhibitors Marine Natural Products

**Figure 4:**
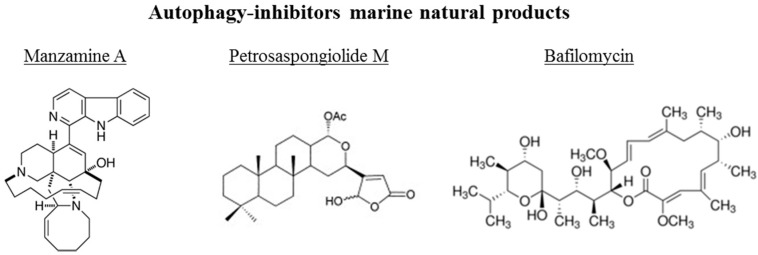
Chemical structure of different autophagy-inhibitors, natural products from marine organisms.

The chemical structures of the marine autophagy-inducers marine natural products are reported in the Figure 4. Manzamine A, a member of the manzamine alkaloids, has been isolated from sponges of the genera *Haliclona* sp., *Xestospongia* sp., and *Pellina* sp. [112,113,114]. It has been reported to have anti-tumor, insecticidal, antibacterial, anti-malarial, and anti-inflammatory activities [115,116,117,118,119]. Recent studies showed that manzamine A has activity against pancreatic cancer cells, decreasing cell dissociation, abrogating cell migration, and sensitizing AsPC-1 pancreatic adenocarcinoma cells towards TRAIL induced apoptosis [120]. Chemogenomic profiling in the yeast *S. cerevisiae* has been performed, demonstrating that manzamine A is an uncoupler of vacuolar ATPases [52]. Manzamine A produced a phenotype very similar to that of the established v-ATPase inhibitor bafilomycin A (toxic macrolide antibiotic derived from *Streptomyces griseus*, inhibitor of autophagosome-lysosome fusion), as revealed by fluorescence microscopy. In pancreatic cancer cells, 10 μM manzamine A affected vacuolar ATPase activity and significantly increased the level of autophagosome marker LC3-II and p62/SQSTM1 as observed by western blot analysis. Combination of manzamine A with bafilomycin A1 did not change the levels of LC3-II when compared to cells treated with bafilomycin A1 alone. These results suggested that this alkaloid could act as a potential inhibitor of autophagy by preventing autophagosome turnover, a promising strategy for the treatment of pancreatic cancer. Since autophagy is essential for pancreatic tumor growth and chemoresistance, targeting this pathway with manzamine A results a promising treatment strategy for pancreatic adenocarcinoma.

The marine γ-hydroxybutenolide terpenoid petrosaspongiolide M has been isolated from a marine sponge, *Petrosaspongia nigra* [121]. It has been demonstrated that this marine compound can exert inhibitory effects on autophagy in human macrophage U937 cells by downregulation of Beclin-1 levels, with anti-inflammatory properties in acute and chronic inflammation [122]. Eight bafilomycins, A1, B1, D, F, G, H, I, and J, purified from *Streptomyces* spp. from marine environments, are potent inhibitors of autophagy as evidenced by experiments using automated microscopy screening assay, which showed autophagosome accumulation [123]. The clasto-lactacystinblactone (LA) or epoxomicin (Epo), two proteinase inhibitors, were recently reported to induce autophagy through inhibition of the PI3K-Akt-mTOR pathway in human retinal pigment epithelial ARPE-19 cells [124]. Using the autophagy inhibitor bafilomycin A1, the protective effects of LA or Epo against menadione-induced oxidative injuries in ARPE-19 cells were reverted.

Seaweeds are very rich in polyphenols [125], which exert potential antitumor activity [126] by inhibiting cancer cell proliferation and metastasis, and promoting tumor regression [21]. Recently, the anti-pancreatic cancer potential of polarity-based polyphenol extractions has been defined from three different seaweeds and their potential molecular targets have been identified [127]. These polyphenols were ethyl acetate fractions of *Hormophysa triquerta*, *Spatoglossum asperum*, and *Padina tetrastromatica*. On this line, Aravindan et al. [127] used a clinically translatable residual pancreatic cancer model to investigate the clinical benefits of seaweed polyphenols in regulating the onset and maintenance of autophagy, particularly in the cells that survive first-line therapy. Human Panc-3.27 and MiaPaCa-2 cells were exposed to these seaweed-derived polyphenols and transcriptional alterations in some autophagy functional regulators (ATG3, ATG5, ATG7, ATG12, LC3A, LC3B, Beclin, Myd88, HMGB1, Rage and TLRs 1–9) were examined by Real Time qPCR. The potential of polyphenols to target ATG3, ATG5, ATG12, LC3A, LC3B, BECN1, and SURIVIN after clinical radiotherapy has been investigated, using a clinically relevant mouse model of residual pancreatic cancer, tissue microarray, and immunohistochemistry procedures. Seaweed polyphenols completely suppressed the transcription of all investigated autophagy regulators in both cell lines. These data suggested that the seaweed-derived polyphenols could serve as effective adjuvants for current pancreatic cells treatments and may inhibit tumor relapse by comprehensively targeting therapy-orchestrated autophagy in residual cells.

## 4. Conclusions

The marine environment harbors a number of macro- and microorganisms that have developed unique metabolic abilities to ensure their survival in diverse and hostile habitats, resulting in the biosynthesis of an array of secondary metabolites with specific activities. Exploration of the sea biodiversity enables the development of new pharmaceuticals with great benefits for human health. Despite the great number of natural products from the marine environment, very few are able to induce and/or inhibit autophagy (summarized in Table 1).

According the data reported in this review, there are no relationships among source species and the isolated molecules, the only exception being alkaloids, which are all derived from sponges (see Table 1). There are no relationships among source species and cancer type in which the compounds are involved. The only exception is represented by autophagy-inducers involved in uterine cancer, all deriving from algae. Finally, there is not a common link among the type of compounds and the type of cancer. For example, the autophagy-inducer alkaloids are able to act on neuroblastoma, leukemia, breast and prostate cancer. The same situation exists for the autophagy-inhibitor compounds. Moreover, based on the studies reported in this review, we could not exclude the possibility that some of these marine compounds may be relevant to other cancer types. Further studies will elucidate this point.

In conclusion, we reported several examples demonstrating that “blue-print autophagy” represents a good tool as an adjuvant for current cancer cell treatments as well as inhibiting tumor relapse.

## Figures and Tables

**Table 1:** Name of compounds, marine organism source, structure, activity as inhibitor or inducer of autophagy, and disease in which they are involved.

Compound	Source	Structure	Autophagy Print	Disease
Monanchocidin A	sponge	alkaloid	Inducer	Leukemia, Prostate Cancer
Clionamines	sponge	aminosteroid	Inducer	Breast cancer
Papuamine	sponge	alkaloid	Inducer	Breast cancer
Rhabdastrellic acid A	sponge	triterpenoid	Inducer	Lung cancer
Stellettin A	sponge	triterpene	Inducer	Melanoma
Xestospongin B	sponge	alkaloid	Inducer	Neuroblastoma
Araguspongine C	sponge	alkaloid	Inducer	Breast cancer
Ilamaquinone	sponge	quinone	Inducer	Colon cancer
Ovothiol A	sea urchin	thiol	Inducer	Liver cancer
Hirsutanol	fungus	sesquiterpene	Inducer	Breast cancer
Xanthocillin X	fungus	diphenol	Inducer	Liver cancer
Salinosporamide A	bacteria	lactone	Inducer	Prostate Cancer
Chromomycin A2	bacteria	polyketide	Inducer	Melanoma
Coibamide A	cyanobacterium	cyclopeptide	Inducer	Glioblastoma
EPA and DHA	algae	fatty acids	Inducer	Lung cancer
Fucoxanthin	algae	carotenoid	Inducer	Uterine Cancer
Methanolic extract	algae	phenol	Inducer	Uterine Cancer
Manzamine A	sponge	alkaloid	Inhibitor	Pancreatic cancer
Petrosaspongiolide	sponge	terpenoid	Inhibitor	Chronic inflammation
Bafilomycin	algae	macrolide antibiotic	Inhibitor	Retinal disease
Polyphenols	algae	phenol	Inhibitor	Pancreatic cancer
